# METTL1-deficient mesenchymal stem cells protect against metabolic-associated fatty liver disease by increasing NAMPT secretion

**DOI:** 10.1093/stcltm/szag016

**Published:** 2026-03-29

**Authors:** Jiang Du, Yuxuan Zhang, Chiheng Wang, Yuyuan Wang, Hongen Zhang, Dunyong Zhao, Juntang Lin

**Affiliations:** Henan Collaborative Innovation Center of Stem Cells and Biotherapy, School of Medical Engineering, Henan Medical University (Xinxiang Medical University), Xinxiang 453003, China; Henan Joint International Research Laboratory of Stem Cell Medicine, School of Medical Engineering, Henan Medical University (Xinxiang Medical University), Xinxiang 453003, China; Henan Collaborative Innovation Center of Stem Cells and Biotherapy, School of Medical Engineering, Henan Medical University (Xinxiang Medical University), Xinxiang 453003, China; Henan Joint International Research Laboratory of Stem Cell Medicine, School of Medical Engineering, Henan Medical University (Xinxiang Medical University), Xinxiang 453003, China; Henan Collaborative Innovation Center of Stem Cells and Biotherapy, School of Medical Engineering, Henan Medical University (Xinxiang Medical University), Xinxiang 453003, China; Henan Joint International Research Laboratory of Stem Cell Medicine, School of Medical Engineering, Henan Medical University (Xinxiang Medical University), Xinxiang 453003, China; Henan Collaborative Innovation Center of Stem Cells and Biotherapy, School of Medical Engineering, Henan Medical University (Xinxiang Medical University), Xinxiang 453003, China; Henan Joint International Research Laboratory of Stem Cell Medicine, School of Medical Engineering, Henan Medical University (Xinxiang Medical University), Xinxiang 453003, China; School of pediatrics, Henan Medical University (Xinxiang Medical University), Xinxiang 453003, China; Department of Gastroenterology, Institute of Digestive Diseases of PLA, The First Affiliated Hospital (Southwest Hospital) of Third Military Medical University (Army Medical University), Chongqing 400038, China; Henan Collaborative Innovation Center of Stem Cells and Biotherapy, School of Medical Engineering, Henan Medical University (Xinxiang Medical University), Xinxiang 453003, China; Henan Joint International Research Laboratory of Stem Cell Medicine, School of Medical Engineering, Henan Medical University (Xinxiang Medical University), Xinxiang 453003, China

**Keywords:** mesenchymal stem cells, metabolic-associated fatty liver disease, METTL1, NAMPT, lipid synthesis

## Abstract

**Background:**

Genetically modified mesenchymal stem cells (MSCs) have been shown to enhance their therapeutic properties, offering more effective treatment options for various diseases, including metabolic associated fatty liver disease (MASLD). The m7G methyltransferase METTL1 plays a critical role in regulating RNA splicing, stability, and translation. This study presents our findings on METTL1 modified human umbilical cord MSCs, emphasizing their therapeutic effects and the mechanisms involved in treating MASLD.

**Methods:**

METTL1 knockdown MSCs were generated via lentiviral shRNA. Key characteristics, including senescence, proliferation, cell cycle, and apoptosis, were assessed in vitro. A high-fat diet (HFD)-induced MASLD mouse model was used to evaluate the effects of MSC transplantation through serological, biochemical, and pathological analyses. Molecular mechanisms were explored using immunofluorescence (IF), Western blotting (WB), and quantitative PCR (qPCR).

**Results:**

Our results indicate that METTL1-deficient MSCs exhibit reduced proliferative capacity and increased susceptibility to senescence and apoptosis. Remarkably, these MSCs significantly decreased lipid accumulation in both in vitro and in vivo MASLD models. We found that METTL1-deficient MSCs secrete higher levels of NAMPT, which activates SIRT1, leading to the inhibition of SREBP1-mediated lipogenic genes. Inhibition of NAMPT reversed the protective effects of METTL1-deficient MSCs against MASLD-related lipid metabolic disorders. Furthermore, overexpression of METTL1 in MSCs exacerbated lipid metabolic disorders in MASLD mice by inhibiting the NAMPT/SIRT1/SREBP1 signaling pathway.

**Conclusion:**

METTL1-deficient MSCs alleviate MASLD-associated lipid metabolic disorders via NAMPT secretion. This suggests that genetically modified MSCs targeting METTL1 may represent a promising therapeutic strategy for the treatment of MASLD.

## Graphical abstract

**Figure:**
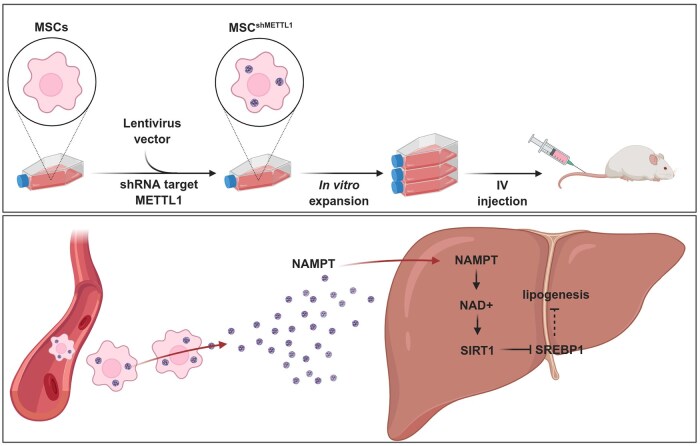

Significance statement Genetically modified mesenchymal stem cells (MSCs) have been shown to enhance their therapeutic properties, offering more effective treatment options for various diseases. Through in vitro and in vivo experiments, we demonstrated that METTL1-deficient MSCs secrete higher levels of NAMPT, activate SIRT1, and inhibit SREBP1-mediated lipogenesis, resulting in a more significant improvement in MASLD. This suggests that targeting METTL1 in transgenic MSCs may represent a promising therapeutic strategy for MASLD.

## Introduction

Metabolic dysfunction-associated steatotic liver disease (MASLD) is characterized by lipid accumulation and degeneration within hepatocytes, progressing through various stages, including simple steatosis, steatohepatitis, liver fibrosis, and ultimately cirrhosis.^1^ In recent years, the prevalence of MASLD has been increasing, representing a significant global health challenge and affecting over 30% of the adult population worldwide.^2^ Currently, only two drugs, Resmetirom and Semaglutide, have received regulatory approval for the clinical management of MASLD.^3^^,^^4^ Ongoing research is aimed at identifying new drug targets and developing innovative therapeutic strategies.^5^ Mesenchymal stem cells (MSCs) have emerged as a promising therapeutic avenue for a range of diseases, including MASLD, due to their ability to secrete cytokines, release extracellular vesicles, and modulate immune responses.^6–8^ Enhancing the survival and secretion capabilities of MSCs is crucial for improving treatment efficacy in MASLD.^9^ Recent advancements have focused on genetic modifications and priming strategies to modify the intrinsic properties of MSCs or to alter their secretion profiles, thereby enhancing their therapeutic potential.^10^ For instance, adipose-derived MSCs with a deficiency in the autophagy-related gene ATG5 have been shown to confer protection against fatty liver disease by increasing the secretion of hepatocyte growth factor (HGF).^11^

N7-methylguanosine (m7G) is a widespread post-transcriptional modification found in eukaryotic RNA, including tRNA, rRNA, and mRNA.^12^ This modification is crucial in the initiation and progression of various diseases.^13^ METTL1 serves as the principal m7G methyltransferase and is involved in both the development and treatment of liver cancer through its regulation of m7G modification in tRNA.^14^^,^^15^ METTL1-deficient hepatocytes exhibit impaired proliferation and reduced regenerative capacity in a partial hepatectomy mouse model. Mechanistically, METTL1 mediates m7G tRNA modifications, which regulate the effects of the Hippo pathway on YAP/TAZ.^16^ Our previous research has demonstrated that METTL1 regulates fatty acid synthesis and oxidation in MASLD through m7G modifications of FoxO1.^17^ These findings indicate that alterations in the METTL1 gene can significantly impact the intrinsic properties of hepatocytes. However, the effects of METTL1 gene modification on the biological characteristics of MSCs and its potential therapeutic implications in various diseases remain largely uninvestigated.

In this study, we utilized lentiviral transfection to create METTL1 knockdown human umbilical cord MSCs and evaluated their biological characteristics. Subsequently, we investigated the therapeutic effects of MSCs with METTL1 gene modification in MASLD and elucidated their potential molecular mechanisms. Our research identifies genetic modification targets for MSC-based therapies in MASLD and provides valuable data to support the application of gene-modified MSCs in the treatment of this condition.

## Methods

### Cell culture and treatment

Human umbilical cord-derived MSCs (CP-CL11) were obtained from Procell (China) and cultured in Dulbecco’s Modified Eagle Medium (DMEM) (Hyclone) supplemented with 10% fetal bovine serum (FBS) (Gibco) and 1% penicillin/streptomycin (Gibco). MSCs at passages 3 to 5 were co-cultured with hepatocytes and subjected to METTL1 gene modification or drug treatment. The modified cells at passages 3-5 were subsequently used for cell transplantation in mice fed a high-fat diet (HFD). Additionally, after treating the cells with fresh medium ­containing the NAMPT inhibitor FK866 (S2799, Selleck, China) for 24 hours, the cells were harvested for subsequent experiments.

Human HepG2 cells (CL-0103) and mouse AML12 cells (CL-0602) were also sourced from Procell (China) and cultured in DMEM supplemented with 10% FBS and 1% penicillin/streptomycin. Free fatty acids (FFAs) were prepared by mixing oleic acid (O1008, Sigma) and palmitic acid (P5585, Sigma) in a 2:1 ratio, dissolved in bovine serum albumin (BSA) solution. Hepatocytes were treated with 400 μM FFAs for 24 hours to establish a lipid accumulation model, followed by subsequent experiments. All cells, including MSCs, were maintained in a cell culture incubator at 37 °C with 5% CO_2_ and in a humidified atmosphere.

### Animals and treatment

The work has been reported in accordance with the ARRIVE guidelines 2.0. All animal experiments were conducted following standard protocols, animal welfare regulations, and the institutional guidelines of Xinxiang Medical University. Male C57BL/6 mice, aged 8 weeks, were obtained from Beijing Vital River Laboratory and housed in a sterile facility under a 12-hour light-dark cycle. To establish the model of MASLD, the mice were fed a HFD for an extended period. This diet consisted of 19.4% protein, 60% fat, and 20.6% carbohydrates (TP23400, Trophic, Jiangsu, China). Mice maintained on a normal chow diet (NCD) (LAD3001M, Trophic, Jiangsu, China) served as the control group. In the stem cell-base therapy experiment, after 8 weeks on the HFD, the mice received their first transplant of MSCs or genetically modified MSCs via tail vein injection, with a dosage of 1 × 10^6^ cells per mouse. A total of two injections were administered, separated by a 3-week interval. Four weeks after the last injection, the mice were euthanized via intraperitoneal injection of sodium pentobarbital (50 mg/kg body weight) for subsequent analysis.

### Lentiviral infections

Lentiviral vectors targeting human METTL1 and control lentiviral vectors containing shRNA targeting GFP were obtained from HanHeng Biotechnology. The specific sequence targeting human METTL1 is: 5′-GCGACTGGATGTGCACTCATTT-3′. The shRNA plasmids were transfected into 293T cells at a specific ratio using Lipofectamine 3000 (Thermo, L3000015, USA). Following a 72-hour incubation period for viral production, lentiviral particles were collected and used to infect MSCs at a 1:1 ratio, resulting in the generation of MSC^shGFP^ and MSC^shMETTL1^ cell lines. Subsequently, puromycin was employed to select and culture the MSCs for further experiments. Human umbilical cord-derived MSCs were infected with lentivirus at passages 3 to 5, and the efficiency of METTL1 expression was evaluated. The modified cells were then transplanted into mice with HFD-induced conditions via tail vein injection.

### Adenoviral infections

Adenoviruses expressing human METTL1 were acquired from HanHeng Biotechnology, along with control adenoviruses containing full-length GFP. Viral replication was performed in laboratory 293A cells. Human umbilical cord-derived MSCs were infected with adenoviruses at passages 3-5, and the effectiveness of METTL1 overexpression was assessed. The engineered cells were then transplanted into HFD-induced mice via tail vein injection.

### Quantitative PCR analysis

Cells and tissues involved in the experiment were subjected to total RNA extraction using TRIzol reagent (DP424, Tiangen, China), adhering to the manufacturer’s instructions. The isolated RNA was then reverse transcribed into cDNA using the PrimerScript RT reagent kit (KR116, Tiangen, China). qPCR experiments and subsequent analyses were conducted using the SYBR Green reagent kit (FP201, Tiangen, China) on the ABI 7500 system. The mRNA expression levels were quantified employing the delta-delta CT method, with GAPDH serving as the internal control for normalization. The primer pairs utilized in this study are specified in Table S1 (see online supplementary material).

### Western blot analysis

Total protein samples were extracted from cells or mouse tissues using RIPA lysis buffer (P0013B, Beyotime, China) supplemented with protease inhibitors (P1005, Beyotime, China) and phosphatase inhibitors (P1045, Beyotime, China). The protein concentration was quantified using a BCA protein assay kit (P0012S, Beyotime, China). Equal amounts of protein samples were loaded onto 10% SDS-polyacrylamide gels for electrophoresis, followed by transfer to PVDF membranes. The membranes were then blocked for 1 hour at room temperature using PBS containing 5% non-fat dry milk to prevent non-specific binding. The membranes were incubated overnight at 4 °C with primary antibodies specific to the target proteins. Following this, the membranes were incubated with HRP-conjugated secondary antibodies. Protein detection was achieved using enhanced chemiluminescence reagents to visualize the antibody-bound proteins. The protein expression levels were analyzed using ImageJ software and normalized to β-tubulin or GAPDH levels to serve as loading controls.

### Co-culture of hepatocytes and MSCs

Hepatocytes, including human HepG2 cells and mouse AML12 cells, were co-cultured with MSCs in a Transwell system (Corning, China). Hepatocytes were seeded in the lower chamber containing DMEM culture medium. Subsequently, MSC^shGFP^ or MSC^shMETTL1^ cells were added to the upper chamber. After a 24-hour co-culture period, the cells were further treated with FFAs for an additional 24 hours. Following these treatments, the hepatocytes in the lower chamber were subjected to Nile Red staining, TG measurement, qPCR analysis, Western blotting, and immunofluorescence (IF) staining to assess the effects of co-culture under FFAs treatment.

### Cell cycle analysis

MSC^shGFP^ or MSC^shMETTL1^ cells were seeded in 25 cm^2^ cell culture dishes. After a 12-hour incubation to promote growth, the cells were treated with FFAs for 24 hours. Then, the cells were fixed with 70% ethanol to maintain their morphology and structural integrity for further analysis. Once fixed, the cells were stained using a cell cycle detection kit (C-1052, Beyotime, China), which allows for the identification of different phases of the cell cycle based on DNA content. The distribution of cells across the various cell cycle phases was then analyzed using a BD FACS flow cytometer. The resulting data were processed and analyzed using FlowJo software.

### Cell apoptosis analysis

MSC^shGFP^ or MSC^shMETTL1^ cells were cultured in 25 cm^2^ cell culture dishes and pre-treated with FFAs for 24 hours. After treatment, the cells were incubated at room temperature for 30 minutes with FITC-Annexin V and propidium iodide (C1062L, Beyotime, China) to identify apoptotic cells. The quantity of apoptotic cells was subsequently quantified using a BD FACS flow cytometer. The resulting data were processed and analyzed using FlowJo software.

### EdU and SA-β-gal staining

MSC^shGFP^ or MSC^shMETTL1^ cells were seeded in a 12-well plate and treated with 400 μM of FFAs. For SA-β-gal Staining: After treatment, the medium was replaced with fresh DMEM and incubated for an additional 3 days. Cell staining was performed using a senescence-associated β-galactosidase (SA-β-gal) staining kit (C0602, Beyotime) according to the manufacturer’s instructions. The presence of blue cytoplasmic staining was used to identify SA-β-gal positive cells, indicating cellular senescence. For EdU Staining: Following SA-β-gal staining, the cells were stained with BeyoClick EdU-555 (C0075S, Beyotime, China) according to the manufacturer’s instructions for 2 hours. Fluorescence was detected and measured using a fluorescence microscope to assess cell proliferation.

### Nile Red staining

Cells were treated with 400 μM FFAs for 24 hours. Subsequently, the cells were fixed with 4% paraformaldehyde (PFA) and stained with Nile Red (Sigma) for 15 minutes in the dark at room temperature. This was followed by DAPI staining for 5 minutes. Images of the stained cells were captured using a confocal laser scanning microscope.

### Histological analysis

Histological analysis was performed following the methods described in previous reports.^8^ Briefly, after deep anesthesia, the mice were euthanized by cervical dislocation. The liver tissues were then fixed in 4% PFA and subsequently embedded in paraffin. Thin sections of 8 μm were cut and stained using hematoxylin and eosin (H&E) staining protocols. For Oil Red O staining, frozen liver sections were initially fixed in 60% isopropanol for 10 minutes. They were then stained with freshly prepared Oil Red O working solution for 30 minutes, followed by rinsing with 60% isopropanol. Afterward, the sections were counterstained with hematoxylin and mounted with an aqueous mounting medium. The histological sections were examined and photographed using an optical microscope.

### Serological analysis

Glucose tolerance tests (GTT) and Insulin tolerance tests (ITT) were conducted as previously described.^8^ Mice were fasted for 16 hours prior to GTT and for 4 hours before ITT. For the GTT, mice received an intraperitoneal injection of glucose (2 g/kg). Blood samples were collected from the tail vein at 0, 15, 30, 60, 90, and 120 minutes post-injection to measure blood glucose levels. For the ITT, mice were administered an intraperitoneal injection of insulin (0.75 U/kg), and blood samples were similarly collected at the aforementioned time points to assess blood glucose levels. Blood glucose levels were monitored using a Sinocare automatic blood glucose meter.

Serum enzyme levels, including alanine aminotransferase (ALT) and aspartate aminotransferase (AST), were determined using specific kits (C009/C010, Jiangsu Jiancheng, China) according to the manufacturer’s instructions. Additionally, triglyceride (TG) and total cholesterol (TC) levels were measured using a commercial kit (A110 and A111, Jiangsu Jiancheng, China), following the manufacturer’s guidelines. The NAD+ content in mouse liver tissues was quantified using a NAD+ assay kit (S0175, Beyotime, China).

### Immunofluorescence

Cells or liver tissues were fixed with 4% PFA. After fixation, the samples underwent blocking and permeabilization to minimize non-specific binding and facilitate antibody penetration. The samples were then incubated with a primary antibody specific to the target antigen. Following thorough washing, a fluorochrome-conjugated secondary antibody was applied for detection. Cell nuclei were stained with DAPI to improve visualization. Finally, images were captured using a confocal laser scanning microscope, providing detailed fluorescence images for further analysis.

### Antibodies

Primary antibodies specific for the following proteins were obtained from Cell Signaling Technology: ACC-1 (#3676), SCD1 (#2794), FASN (#3180), p-AKT (#4060), and AKT (#9272). Additionally, a primary antibody to METTL1 (14994-1-AP), SIRT1 (13161-1-AP), NAMPT (11776-1-AP) and SREBP1 (14088-1-AP) were purchased from Proteintech. Primary antibodies to SREBP1 (abs131802), β-actin (abs132001), and β-tubulin (abs171597) were purchased from absin. In Western Blotting experiments, secondary antibodies goat anti-rabbit-IgG (#abs20040) and goat anti-mouse-IgG (#abs20039) from absin were utilized to detect primary antibody-bound target proteins. For Immunofluorescence analysis, CoraLite488-conjugated Goat Anti-Rabbit IgG (#SA00013-2) and CoraLite594-conjugated Goat Anti-Mouse IgG (#SA00013-3) secondary antibodies were used, both sourced from Proteintech.

### Transcriptomic and secretomic analysis

For the transcriptomic analysis of MSCs, cells from the MSC^shGFP^ and MSC^shMETTL1^ groups were cultured in serum-free conditions for 48 hours, after which the cells were collected for RNA extraction. The extracted RNA samples were subsequently used for transcriptomic analysis (APTBIO, Shanghai, China). The experimental workflow included RNA extraction, quality assessment, library preparation, sequencing, and bioinformatics analysis.

For the secretomic analysis of MSCs, the supernatants from the MSC^shGFP^ and MSC^shMETTL1^ groups were collected after 48 hours of serum-free culture. Quantitative analysis of the secreted proteins was performed using Data-Independent Acquisition (DIA) mass spectrometry (APTBIO, Shanghai, China). In summary, the experimental workflow consisted of protein extraction, peptide digestion, liquid chromatography-tandem mass spectrometry (LC-MS/MS) for DIA data acquisition, database searching, qualitative and quantitative result analysis, and bioinformatics evaluation.

### ELISA assays

The conditioned media from MSC^shGFP^ or MSC^shMETTL1^ cells were concentrated 10-fold using ultrafiltration. Subsequently, the expression levels of NAMPT, SOD2, PCSK9, LDH, and PRDX1 in the supernatants were measured using ELISA (Enzyme-Link, Shanghai, China), following the manufacturer’s guidelines.

### Statistical analysis

Statistical analysis was performed using GraphPad Prism software. The normality of the data was assessed using the Kolmogorov-Smirnov test. If the data were not normally distributed, the Kruskal–Wallis test followed by Dunn’s multiple comparisons test was employed for comparing multiple groups. Conversely, if the data were found to be normally distributed, the Brown-Forsythe test was used to assess homogeneity of variance. For comparisons between multiple groups, one-way ANOVA was conducted, followed by Tukey’s post hoc test. GTT and ITT were analyzed using two-way ANOVA. Paired comparisons were performed using an unpaired Student’s *t*-test. All experiments were independently repeated at least three times. Quantitative data are presented as means ± SEM, and significance levels are indicated in the figures, with three decimal places retained.

## Results

### METTL1-deficient MSCs exhibit reduced proliferation and increased susceptibility to senescence and apoptosis

**Figure 1.**
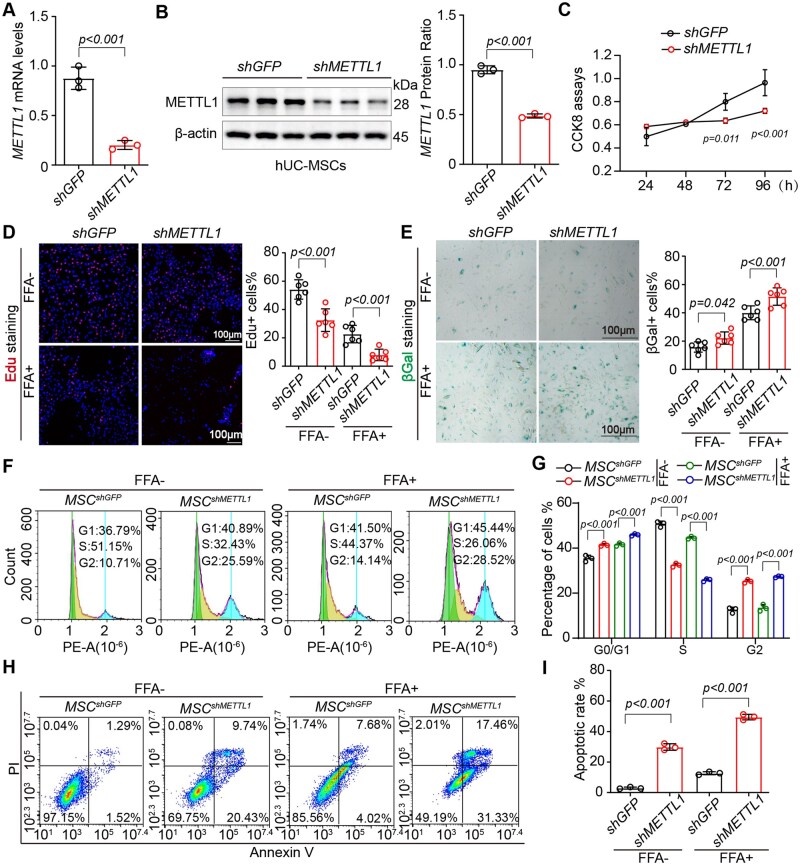
METTL1-deficient MSCs display reduced proliferation and increased susceptibility to senescence and apoptosis. (A, B) Evaluation of METTL1 mRNA and protein expression in MSCs transduced with lentiviruses containing shRNA targeting METTL1. (C) CCK-8 assays comparing the proliferation of MSC^shGFP^ and MSC^shMETTL1^. (D) Representative images of EdU staining in MSC^shGFP^ and MSC^shMETTL1^ cells were captured following a 24-hour incubation with FFA (scale bars = 100 μm). (E) Representative images of SA-β-gal staining were performed using a senescence β-galactosidase staining kit in the indicated groups (scale bars = 100 μm). (F, G) Cell cycle analysis of MSC^shGFP^ and MSC^shMETTL1^, presented for the G0/G1, S, and G2/M phases in the respective groups. (H, I) Analysis of cell apoptosis was conducted in MSC^shGFP^ and MSC^shMETTL1^under conditions of FFA exposure or without. All statistical analyses are presented in the right panel of the data. For all statistical plots, data are expressed as mean ± S.E.M, and statistical significance is indicated in the figure.

To evaluate the role of the m7G methyltransferase METTL1 in the biological characteristics of MSCs, we employed lentiviral infection technology to knock down METTL1 in umbilical cord-derived MSCs, designated as MSC^shMETTL1^. As illustrated in the figures, METTL1 expression at both RNA and protein levels was significantly decreased in MSC^shMETTL1^ compared to control cells (MSC^shGFP^) (Figure 1A, B). Subsequent CCK-8 assays revealed that MSC^shMETTL1^ exhibited lower proliferation rates and reduced cell viability (Figure 1C). To further investigate the effects of METTL1 deficiency on MSC characteristics, we treated the cells with FFAs. The results demonstrated that MSC proliferation was significantly inhibited after FFAs treatment, with clear signs of cellular senescence. Notably, MSC^shMETTL1^ displayed an even further reduced proliferation rate and a more pronounced senescence phenotype, as confirmed by 5-ethynyl-2'-deoxyuridine (EdU) and β-galactosidase (β-gal) staining (Figure 1D, E). Moreover, flow cytometry analysis indicated that the cell cycle of MSC^shMETTL1^ was arrested in the G0 phase compared to MSC^shGFP^ (Figure 1F, G). Additionally, MSC^shMETTL1^ showed a heightened propensity for apoptosis, regardless of FFAs treatment (Figure 1H, I). These findings suggest that the absence of METTL1 impairs MSC proliferation, increases their susceptibility to aging and apoptosis, and ultimately leads to compromised biological characteristics.

### METTL1-deficient MSCs inhibit lipid synthesis in hepatocytes

**Figure 2.**
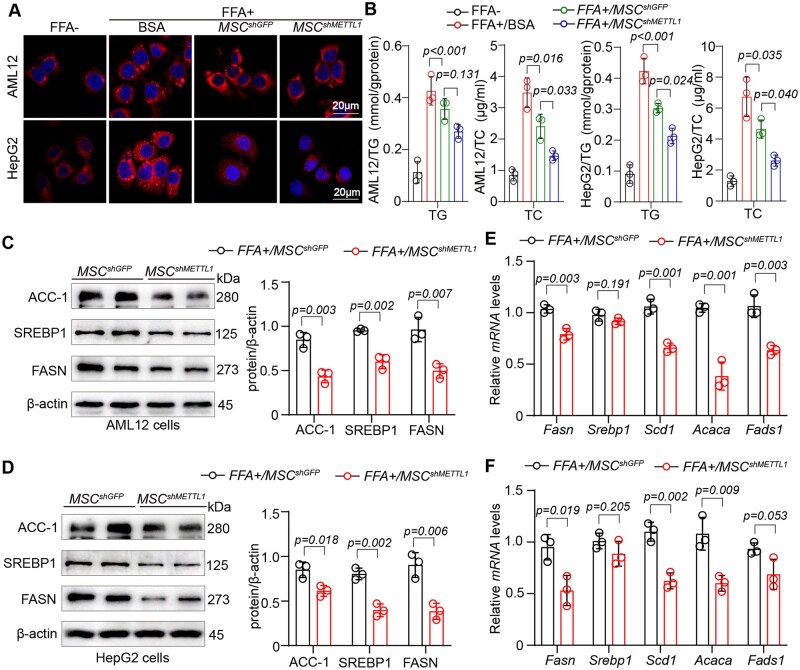
METTL1-deficient MSCs inhibit lipid synthesis in hepatocytes. (A) Representative images of Nile Red staining in hepatocytes co-cultured with MSC^shGFP^ and MSC^shMETTL1^ following treatment with FFA (Scale bar = 20 μm). (B) Measurement of TG content in hepatocytes in the indicated groups. (C, D) Western blot analysis of lipid metabolism-related gene expression (FASN, SREBP1, SCD1) in the indicated groups. (E, F) qPCR analysis of lipid synthesis gene expression (*Fasn, Scd1, Srebp1, Fads1* and *Acaca*) in AML12 and HepG2 cells co-cultured with MSC^shGFP^ and MSC^shMETTL1^. For all statistical graphs, data are presented as mean ± S.E.M, with statistical significance is indicated in the figure.

MSC^shMETTL1^ exhibited compromised biological characteristics compared to MSC^shGFP^. However, the impact of these METTL1-defective MSCs on MASLD remains unclear. To investigate the role of MSC^shMETTL1^ in regulating lipid metabolism in hepatocytes, we established an in vitro co-culture system of MSCs and hepatocytes. Notably, MSC^shMETTL1^ significantly reduced lipid content in hepatocytes exposed to FFAs treatment, as demonstrated by Nile red staining, triglyceride (TG) assays, and cholesterol measurements (Figure 2A, B).

Furthermore, we observed that MSC^shMETTL1^ markedly decreased the expression of lipid synthesis-related genes (FASN, SREBP1, and ACC-1) at the protein level compared to MSC^shGFP^ (Figure 2C, D). Similarly, co-culture with MSC^shMETTL1^ inhibited the expression of lipid synthesis-related genes (*Fasn*, *Scd1*, *Acaca* and *Fads1*), except for *Srebp1* (Figure 2E, F). These findings suggest that, although METTL1-deficient MSCs is more susceptible to senescence and apoptosis, it may have a pronounced effect in reducing lipid accumulation in hepatocytes *in vitro*.

### METTL1-deficient MSCs transplantation attenuates MASLD-related metabolic disorders

**Figure 3.**
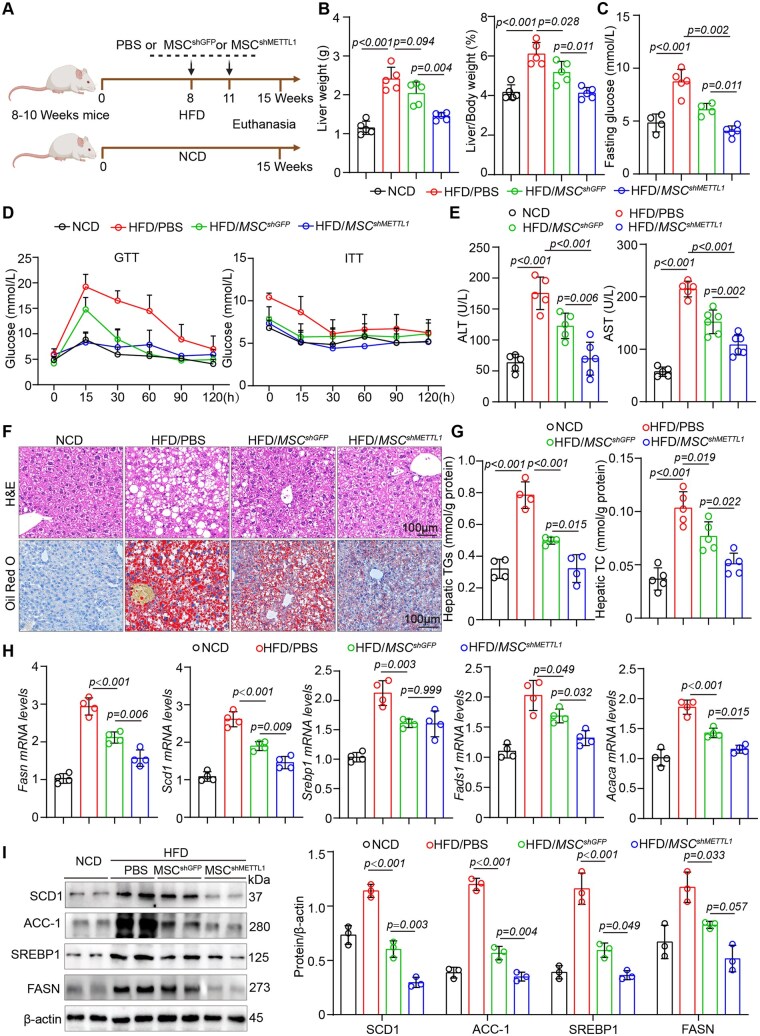
Transplantation of METTL1-deficient MSCs alleviates metabolic disorders associated with MASLD. (A) Schematic diagram of the animal experiment. (B) Evaluation of liver weight and the liver-to-body weight ratio in the indicated mice. (C) Assessment of fasting blood glucose levels in the indicated mice. (D) Analysis of GTT and ITT for the indicated groups. (E) Measurement of serum ALT and AST levels following 7 weeks of cell transplantation. (F) Representative images of HE and Oil Red O staining for analysis of mouse liver tissue (Scale bar = 100 μm). (G) Determination of TG and TC levels in the liver tissue of the specified mice. (H) qPCR analysis of lipid synthesis-related genes, including *Fasn, Scd1, Srebp1, Fads1*, and *Acaca* in the specified groups. (I) Western blot analysis of lipid metabolism-related proteins in the specified groups. For all statistical graphs, individual data points represent individual mice, and data are presented as mean ± S.E.M. Statistical significance is indicated as shown in the figure.

To further investigate the effects of METTL1-deficient MSC transplantation on lipid metabolism, we employed a HFD-induced fatty liver mouse model to evaluate the therapeutic potential of MSCs (Figure 3A). After 15 weeks of HFD feeding, the mice displayed significant increases in body weight, liver-to-body weight ratio, and fasting blood glucose levels. Notably, transplantation of METTL1-deficient MSCs resulted in a more pronounced reduction in body weight, liver-to-body weight ratio, and fasting blood glucose levels compared to MSC^shGFP^ (Figure 3B, C and Figure S1A—see online supplementary material for a color version of this figure). However, changes in food intake were minimal, regardless of whether the mice received control MSCs or METTL1-deficient MSC transplantation (Figure S1B—see online supplementary material for a color version of this figure). GTTs and ITTs revealed that transplantation of MSC^shMETTL1^ significantly restored insulin sensitivity compared to MSC^shGFP^ (Figure 3D). We further investigated the protective effects of METTL1-deficient MSCs on liver function. Our results indicated that, compared to control MSCs, transplantation of MSC^shMETTL1^ led to a significant decrease in serum ALT and AST levels in HFD-fed mice (Figure 3E). These findings suggest that METTL1-deficient MSCs have a greater capacity to ameliorate HFD-induced liver dysfunction and insulin resistance.

Biochemical and histological analyses demonstrated that, compared to mice fed an NCD, serum and liver TG were significantly elevated in HFD-fed mice. Transplantation of MSC^shMETTL1^ significantly improved liver tissue morphology compared to control MSCs and reduced serum and liver lipid accumulation in HFD-fed mice, as evidenced by HE staining, Oil Red O staining, TG and TC assays (Figure 3F, G and Figure S1C—see online supplementary material for a color version of this figure). Additionally, we found that MSC^shMETTL1^ significantly inhibited the expression of genes involved in fatty acid synthesis (*Fasn*, *Scd1*, *Acaca,* and *Fads1*) in the livers of HFD-induced MASLD mice at the transcriptional level; however, the effect on *Srebp1* was minimal (Figure 3H). At the protein level, transplantation of MSC^shMETTL1^ significantly inhibited the expression of lipogenic genes (FASN, SCD1, and ACC1), including SREBP1(Figure 3I). These data suggest that METTL1-deficient MSCs are more effective in inhibiting lipid accumulation.

### METTL1-deficient MSCs exhibit elevated NAMPT secretion

**Figure 4.**
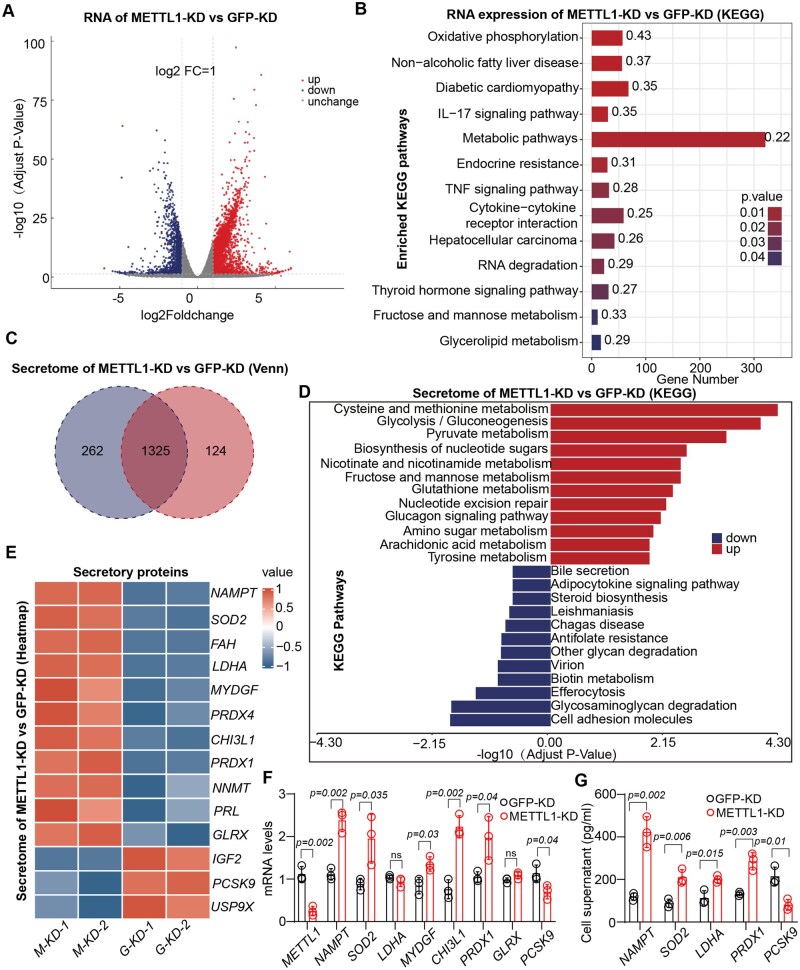
METTL1-deficient MSCs exhibit elevated NAMPT secretion. (A) Volcano plot depicting differentially expressed genes between MSC^shGFP^ and MSC^shMETTL1^, as analyzed by RNA sequencing. (B) Identification of genes significantly enriched in GO analysis for MSC^shMETTL1^ cells. (C) Venn diagram illustrating the distinct proteins present in the secretome of MSC^shGFP^ and MSC^shMETTL1^. (D) GO analysis of proteins that are significantly enriched in the secretome of MSC^shMETTL1^ cells. (E) Heatmap representation of differentially expressed secretory proteins implicated in the regulation of lipid metabolism associated with MASLD in MSC^shGFP^ and MSC^shMETTL1^. (F) qPCR analysis of the expression of relevant genes in the specified cells. (G) ELISA measurements of the levels of differentially expressed secretory proteins in the supernatants of MSC^shGFP^ and MSC^shMETTL1^ cells. For all statistical graphs, individual data points represent independent experimental replicates, and data are presented as mean ± S.E.M. Statistical significance is indicated as shown in the figure.

MSCs exhibit significant secretory capabilities and can ameliorate various diseases through their paracrine functions.^18^ To further elucidate the molecular mechanisms by which METTL1-deficient MSCs more effectively alleviate lipid metabolic disorders in MASLD, we conducted a comparative analysis of RNA expression and the secretome of METTL1-deficient MSCs versus control MSCs. RNA sequencing revealed a total of 4135 differentially expressed genes (DEGs), with 2649 genes upregulated and 1486 genes downregulated (Figure 4A). Gene Ontology (GO) enrichment analysis indicated that the majority of these differentially expressed genes were associated with metabolic signaling pathways and non-alcoholic fatty liver disease (Figure 4B). Additionally, we conducted a comparative analysis of the secretome of METTL1-deficient MSCs and control MSCs using quantitative proteomics. The results revealed that 1325 proteins were identified in both groups, primarily involved in pathways related to cellular glucose and lipid metabolism (Figure 4C, D). Moreover, our differential protein analysis indicated that proteins such as NAMPT, SOD2, FAH, and LDHA, which confer protective effects against MASLD, were secreted in greater quantities by METTL1-deficient MSCs. In contrast, proteins such as PCSK9 and IGF2 were secreted at lower levels (Figure 4E). These findings suggest that, from the perspective of MSC secretomics, METTL1-deficient MSCs may be more effective in ameliorating metabolic dysfunction associated with MASLD. Subsequently, we conducted qPCR and ELISA assays on these secretory factors, revealing that METTL1 deficiency significantly enhanced the expression of NAMPT at both the RNA and secreted protein levels (Figure 4F, G). This indicates that NAMPT secreted by METTL1-deficient MSCs may play a crucial role in improving MASLD.

### NAMPT/SIRT1/SREBP1 mediates the protective effects of METTL1-deficient MSCs in MASLD

**Figure 5.**
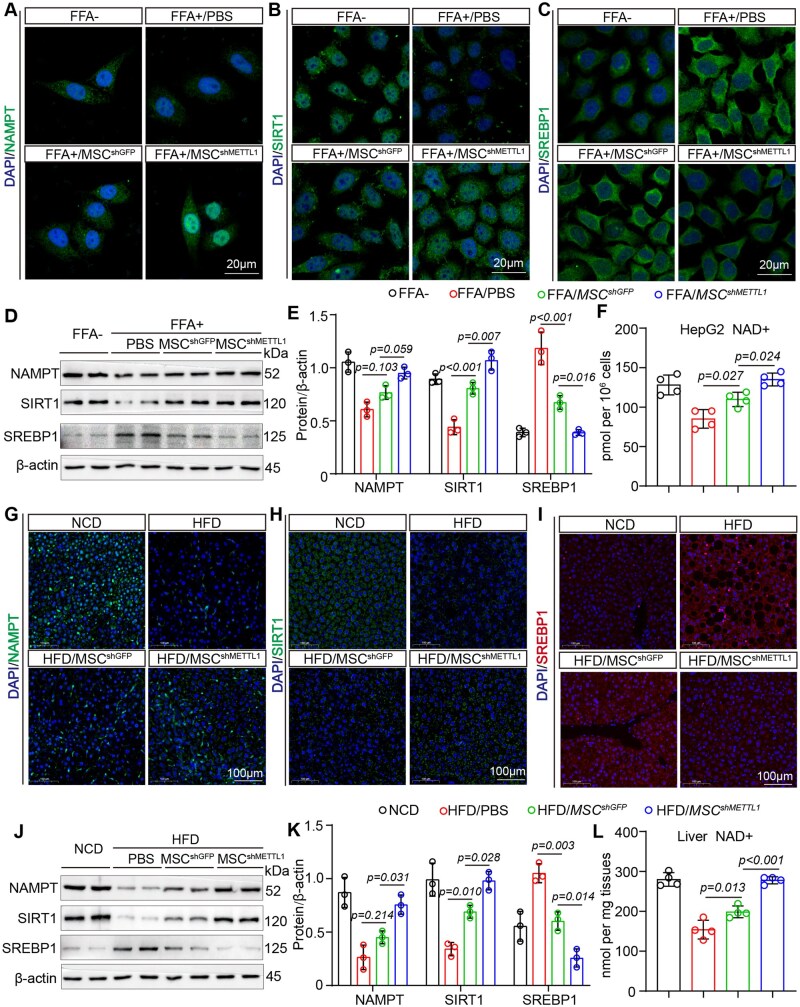
NAMPT/SIRT1/SREBP1 mediates the protective effects of METTL1-deficient MSCs in MASLD. (A-C) Representative images of IF staining for NAMPT, SIRT1 and SREBP1 in HepG2 cells co-cultured with MSC^shGFP^ and MSC^shMETTL1^ cells after treatment with FFA (Scale bar = 20 μm). (D, E) Western blot analysis of NAMPT, SIRT1 and SREBP1 expression in HepG2 cells co-cultured with MSC^shGFP^ and MSC^shMETTL1^ cells following FFA treatment. (F) The NAD+ content was measured in the indicated cells. (G-I) Representative images of IF staining for NAMPT, SIRT1 and SREBP1 in liver tissues from mice transplanted with MSC^shGFP^ and MSC^shMETTL1^ cells after 16 weeks of HFD feeding (Scale bar = 100 μm). (J, K) Western blot analysis of NAMPT, SIRT1, and SREBP1 expression in liver tissues from mice transplanted with MSC^shGFP^ and MSC^shMETTL1^ cells after 16 weeks of HFD feeding. (L) The NAD+ content was measured in the indicated mouse liver tissues. For all statistical graphs, data are presented as mean ± S.E.M., with statistical significance indicated in the figure.

NAMPT regulates the NAD+ pool, thereby influencing cellular oxidative stress responses, apoptosis, lipid and glucose metabolism, inflammation, and insulin resistance in the context of obesity and related diseases.^19^ Previous studies have demonstrated that NAMPT can also modulate lipid metabolism via SIRT1.^20^ To investigate these effects, we conducted IF, WB analysis, and measurements of NAD+ content in hepatocytes within a co-culture system. Our findings revealed that FFA stimulation significantly decreased the expression of NAMPT and reduced NAD+ levels. MSC^shGFP^ enhanced NAMPT expression and NAD+ levels in hepatocytes; however, treatment with METTL1-deficient MSCs further amplified these effects (Figure 5A, D-F). Additionally, we evaluated the expression levels of SIRT1 and SREBP1. The results indicated that treatment with METTL1-deficient MSCs significantly upregulated SIRT1 expression while downregulating SREBP1 expression, suggesting a role in inhibiting lipid synthesis gene expression (Figure 5B-D).

Furthermore, we also performed IF, WB, and NAD+ content assessments on mice that received MSC transplants. The results demonstrated that transplantation of MSC^shMETTL1^ significantly increased the levels of NAMPT and SIRT1, while decreasing the expression of SREBP1 in the liver compared to those receiving MSC^shGFP^ (Figure 5G-K). Additionally, NAD+ levels in the livers of mice receiving METTL1-deficient MSCs were elevated, which enhanced cellular metabolism (Figure 5L). These findings indicate that NAMPT secreted by METTL1-deficient MSCs regulates lipid synthesis in MASLD by modulating the SIRT1/SREBP1 signaling pathway.

### The protective effect of METTL1-deficient MSCs on MASLD related metabolism disorder depends on the secretion of NAMPT

**Figure 6.**
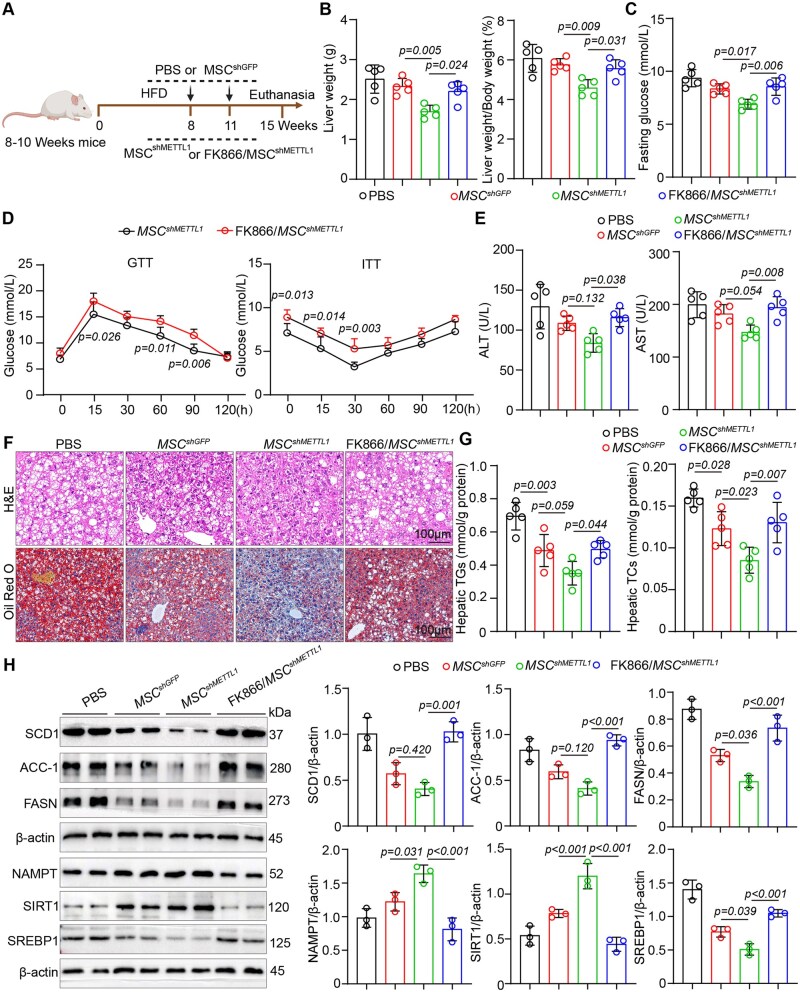
FK866 primed METTL1-deficient MSCs fail to protect against MASLD-related metabolic disorders due to impaired NAMPT secretion. (A) Schematic diagram of the animal experiment. (B) Assessment of liver weight and the liver-to-body weight ratio in the indicated mice. (C) Evaluation of fasting blood glucose levels in the specified mice. (D) GTT and ITT analyses were conducted on the designated groups. (E) Measurement of serum ALT and AST levels was conducted 7 weeks post-cell transplantation. (F) Representative images of HE and Oil Red O staining of mouse liver tissues (Scale bar = 100 μm). (G) Determination of TG and TC levels in the liver tissues. (H) Western blot analysis was performed to investigate the proteins associated with lipid metabolism and NAMPT/SIRT1 signaling in mouse liver tissues. For all statistical graphs, individual data points represent individual mice, and data are presented as mean ±S.E.M. Statistical significance is indicated as shown in the figure.

To further investigate the critical role of NAMPT in the protective effects of MSC^shMETTL1^ against MASLD, we treated MSCs with the NAMPT inhibitor FK866 to block NAMPT expression. Our findings demonstrated that FK866 effectively suppressed both the expression and secretion of NAMPT in MSCs, as validated by WB and ELISA (Figure S2A, B—see online supplementary material for a color version of this figure). Additionally, FK866 treatment in hepatocytes promoted lipid synthesis mediated by SIRT1/SREBP1 signaling while reducing the levels of NAD+ within the liver cells (Figure S3A, B—see online supplementary material for a color version of this figure). We then transplanted MSC^shGFP^, MSC^shMETTL1^, and FK866-primed MSC^shMETTL1^ into mice fed a HFD. After 15 weeks on this diet, we assessed the expression of NAMPT and the levels of NAD+ in the liver tissues of the mice (Figure 6A). The results indicated that transplantation of FK866-treated MSC^shMETTL1^ significantly reduced both NAMPT expression and NAD+ levels in the liver compared to MSC^shMETTL1^ transplantation, indicating that FK866 effectively inhibits NAMPT delivery from MSCs *in vivo* (Figure 6H and Figure S4D—see online supplementary material for a color version of this figure).

Furthermore, we found that FK866-primed MSC^shMETTL1^ did not affect food intake or body weight changes compared to MSC^shMETTL1^ (Figure S4A, B—see online supplementary material for a color version of this figure). However, transplantation of FK866-primed MSC^shMETTL1^ led to an increased liver-to-body weight ratio and elevated fasting blood glucose levels compared to MSC^shMETTL1^ (Figure 6B, C). GTT and ITT assays indicated that transplantation of FK866-primed MSC^shMETTL1^ exacerbated insulin resistance in HFD mice compared to MSC^shMETTL1^ (Figure 6D). Additionally, liver aminotransferase assays revealed that serum levels of ALT and AST were higher following the transplantation of FK866-primed MSC^shMETTL1^ compared to MSC^shMETTL1^ (Figure 6E).

Consistent with previous findings, the transplantation of MSC^shMETTL1^ significantly reduced lipid accumulation in MASLD mice. However, compared to MSC^shMETTL1^, the transplantation of FK866-treated MSC^shMETTL1^ resulted in a marked increase in lipid accumulation, as confirmed by biochemical and histological analyses (Figure 6F, G and Figure S4C—see online supplementary material for a color version of this figure). At the molecular level, we observed that transplantation of FK866-primed MSC^shMETTL1^ increased the expression levels of factors associated with lipid synthesis compared to MSC^shMETTL1^. Additionally, SIRT1 expression decreased following the transplantation of FK866-primed MSC^shMETTL1^, while SREBP1 expression increased (Figure 6H). These results suggest that the protective effects of METTL1-deficient MSCs against liver lipid accumulation in MASLD mice depend on the secretion of NAMPT.

### METTL1-overexpressing MSCs accelerate MASLD progression with reduced NAMPT secretion

**Figure 7.**
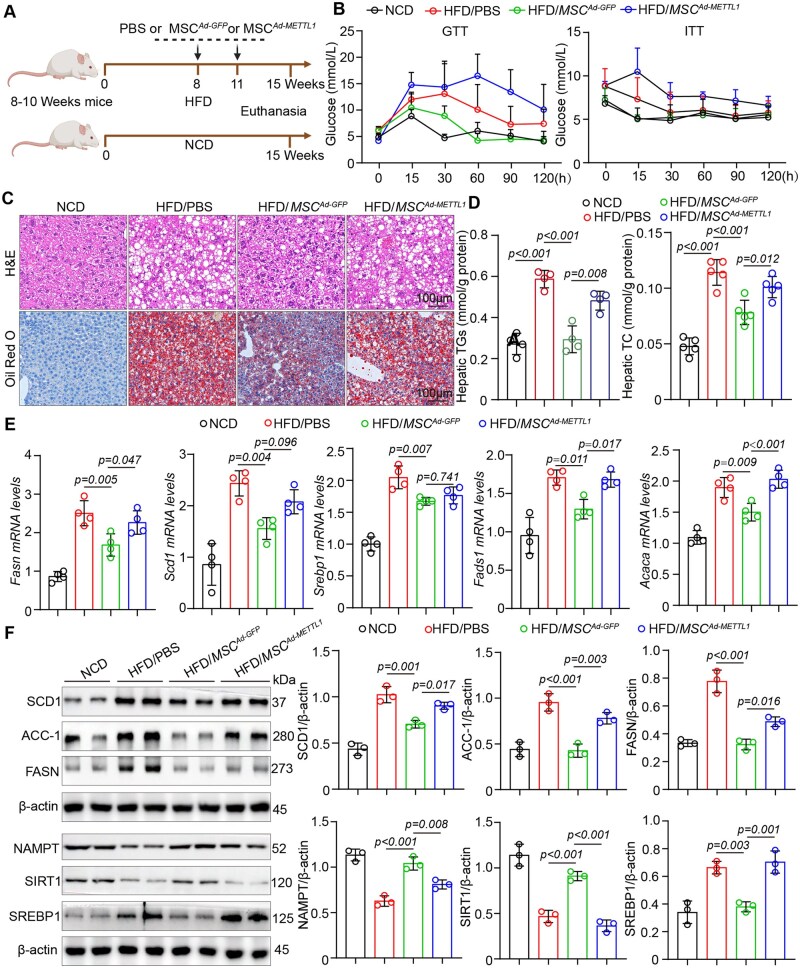
METTL1-overexpressing MSCs accelerate MASLD progression with reduced NAMPT secretion. (A) Schematic diagram of the animal experiment. (B) GTT and ITT analyses were conducted on the designated groups. (C) Representative images of HE and Oil Red O staining of mouse liver tissues (Scale bar = 100 μm). (D) Measurement of TG and TC levels in the liver tissues. (E) qPCR analysis of lipid synthesis-related genes in the designated groups. (F) Western blot analysis was performed to investigate the proteins associated with lipid metabolism and NAMPT/SIRT1 signaling in mouse liver tissues. For all statistical analyses, individual data points represent individual mice, and data are presented as mean ±S.E.M. Statistical significance is indicated as shown in the figure.

To explore the role of METTL1-overexpressing MSCs in the context of MASLD, we engineered adenoviruses to enable the overexpression of METTL1 in MSCs. These modified cells were subsequently transplanted into mice subjected to a HFD to monitor the progression of MASLD (Figure 7A). Our results indicated that, compared to the control group receiving MSC^AdGFP^, the mice that received METTL1-overexpressing MSCs exhibited no significant changes in both body weight and food intake (Figure S5A, B—see online supplementary material for a color version of this figure). Notably, there was a significant increase in the liver-to-body weight ratio and fasting blood glucose levels in these mice (Figure S5C, D—see online supplementary material for a color version of this figure). Additionally, serum transaminase levels indicated that the transplantation of METTL1-overexpressing MSCs led to ­elevated ALT and AST levels in the MASLD mice compared to those receiving control MSCs (Figure S5E—see online ­supplementary material for a color version of this figure). GTT and ITT assays further demonstrated that METTL1-overexpressing MSC transplantation exacerbated insulin resistance relative to the MSC^adGFP^ group (Figure 7B). These findings suggest that the transplantation of MSCs with METTL1 overexpression accelerates liver dysfunction and insulin resistance.

Biochemical and histological analyses demonstrated that the transplantation of METTL1-overexpressing MSCs significantly enhanced serum and liver lipid accumulation in mice fed a HFD, as evidenced by HE staining, Oil Red O staining, TG and TC assays (Figure 7C, D and Figure S5F—see online supplementary material for a color version of this figure). Additionally, we observed increased transcription and protein levels of fatty acid synthesis-related genes, such as FASN, SCD1, and FADS1, following the transplantation of these MSCs (Figure 7E, F). Concurrently, levels of NAMPT and SIRT1 in the livers of the METTL1-overexpressing MSC recipients were found to be decreased, while SREBP1-mediated lipid synthesis was enhanced (Figure 7F). These results suggest that METTL1-overexpressing MSCs may reduce NAMPT secretion, thereby promoting SREBP1-mediated lipid synthesis in MASLD.

## Discussion

MSC-based therapy is emerging as a promising approach for treating a variety of diseases and injuries. However, several challenges persist, including low survival rates, low homing efficiency, and reduced implantation rates, which collectively limit the therapeutic efficacy of transplanted MSCs.^21^ Various strategies have been developed to enhance the therapeutic potential of MSCs, including preconditioning, biomaterial modification, and genetic engineering.^22^ Genetically modified MSCs can enhance their survival duration, biological properties, and secretion profiles, thus contributing to disease treatment.^23^ The therapeutic efficacy of MSCs is generally considered to be correlated with their physiological status. For instance, MSCs overexpressing the Exendin-4 gene exhibit increased cell proliferation, enhanced resistance to cellular senescence and apoptosis, and improved therapeutic outcomes in diabetic mouse models.^24^ Conversely, MSCs deficient in ATG5 have been shown to promote cell survival and improve lipid metabolism disorders in mice with MASLD.^11^^,^^25^ In our study, we observed that METTL1-deficient MSCs demonstrated reduced proliferation and increased susceptibility to senescence and apoptosis. Notably, these METTL1-deficient MSCs effectively inhibited excessive lipid synthesis in MASLD both in vivo and in vitro. However, the survival of MSCs post-transplantation is often transient; they typically undergo apoptosis and are subsequently phagocytosed by host cells.^26^ Increasing evidence suggests that certain therapeutic effects of MSCs, particularly in immune modulation and tissue repair, may actually be initiated through their apoptosis or phagocytosis processes.^27–29^ Previous studies have indicated that MSCs must undergo rapid cell death in vivo to exert optimal anti-inflammatory effects.^27^ Following the engulfment of apoptotic MSCs by recipient phagocytes, these phagocytes produce indoleamine 2,3-dioxygenase, a crucial factor for achieving immunosuppression.^30^ These findings suggest that the vitality of MSCs may not directly correlate with their therapeutic efficacy. Instead, the transplantation of MSCs that are close to apoptosis may yield better therapeutic outcomes in certain diseases, largely depending on the specific bioactive substances they release.

RNA sequencing and secretomic analysis of MSCs with METTL1 knockdown revealed an upregulation of several factors, including NAMPT, SOD2, FAH, and LDHA, all of which have protective effects against MASLD, with NAMPT showing particularly significant increases. NAMPT serves as a key rate-limiting enzyme in NAD synthesis in mammals, influencing the activity of NAD-dependent enzymes and thereby regulating cellular metabolism.^31^ Research has demonstrated that decreased NAMPT activity is linked to cellular aging.^32^ In fact, many studies have reported that physiological aging is associated with a systemic decline in circulating NAMPT levels, and supplementation with young NAMPT can reverse age-related functional decline.^32^ Additionally, MSCs from young mice contain higher levels of NAMPT compared to those from older mice.^15^ These findings establish a general principle that “aging leads to decreased NAMPT secretion.” However, it is intriguing that in our study, while METTL1 deficiency resulted in cellular aging, it also led to increased secretion of NAMPT.

Distinguishing whether the elevated secretion of NAMPT we observed is a direct molecular consequence of METTL1 deficiency or merely a passive phenomenon associated with cellular senescence is indeed significant. Cellular senescence is typically accompanied by alterations in the secretory phenotype, known as the senescence-associated secretory phenotype (SASP), which may include various factors. Our secretomics analysis revealed that MSCs lacking METTL1 do not upregulate all SASP-related factors. Instead, their secretome exhibits selective changes: among the numerous proteins analyzed, only a few protective factors, such as NAMPT and SOD2, were significantly upregulated. This pattern of “selective high secretion of beneficial factors” stands in stark contrast to the typical SASP characterized by the massive release of pro-inflammatory factors, suggesting the existence of specific regulatory pathways rather than a mere byproduct of the aging process. In our study, we found that the absence of METTL1 led to an increased susceptibility of MSCs to aging, yet these cells were capable of secreting higher levels of NAMPT, presenting an intriguing paradox. This suggests that METTL1 deficiency may reprogram the secretory profile of MSCs, pushing them into a unique functional state where they exhibit a senescent-like phenotype while still being capable of selectively high secretion of beneficial factors such as NAMPT. This “therapeutic secretory phenotype” is likely a proactive and specific effect resulting from genetic modification, rather than a passive consequence of aging. Furthermore, literature has reported that METTL1 deficiency can lead to cellular senescence, which aligns with our conclusions.^33^ Thus, we speculate that the relationship between METTL1, NAMPT secretion, and cellular senescence may represent independent phenotypes. Further investigation is necessary to elucidate these mechanisms in future research.

METTL1 is an essential methyltransferase responsible for the m7G modification of tRNA, mRNA, and other types of RNA, thus influencing RNA stability and protein expression levels. In this study, we observed an upregulation of NAMPT secretion resulting from METTL1 deficiency through secretomics analysis. However, the precise mechanism by which METTL1 deficiency impacts NAMPT expression and secretion remains unclear, which is crucial for understanding our research. Multiple studies have reported that NAMPT expression decreases in aging cells, while our findings indicate that MSCs lacking METTL1 exhibit a senescent phenotype alongside increased NAMPT secretion and expression. This suggests that the regulation of NAMPT secretion by METTL1 is not solely due to its role in mediating cellular senescence, but rather reflects a direct or indirect function of METTL1 deficiency. We hypothesize that the loss of METTL1 may lead to a reduction in the m7G modification levels of certain upstream inhibitory factors, resulting in decreased expression of these factors and subsequently promoting NAMPT expression. Additionally, METTL1 deficiency may enhance vesicular sorting, accelerating the recycling of endosomes containing NAMPT to the cell membrane, thereby increasing NAMPT release.^11^

Genetic deficiency of liver NAMPT exacerbates dyslipidemia and liver injury in mice fed a HFD.^34^ Additionally, the inhibition of NAMPT using the inhibitor FK866 worsens obesity induced by the HFD in mice, suggesting that hepatocyte NAMPT could serve as a potential target for preventing dyslipidemia and fatty liver disease.^35^ Our study further indicates that MSCs lacking METTL1 can ameliorate lipid metabolic disorders induced by HFD. However, treatment of METTL1-deficient MSCs with FK866 abolishes their protective effect against these lipid metabolic disorders, demonstrating that METTL1-deficient MSCs improve MASLD primarily through increased secretion of NAMPT. SIRT1, an NAD-dependent histone deacetylase, is positively regulated by NAMPT and has been extensively reported to play a role in lipid metabolism regulation.^36^^,^^37^ Activation of the NAMPT/NAD+/SIRT1 axis significantly alleviates inflammation and lipid metabolic disorders in the livers of MASLD mice.^38^ Furthermore, it has been reported that SIRT1 can directly deacetylate SREBP1, thereby inhibiting SREBP1 expression through post-translational modifications that regulate lipid synthesis.^39^^,^^40^ Our data suggest that METTL1-deficient MSCs enhance NAMPT secretion, which subsequently inhibits SREBP1-mediated lipid synthesis in MASLD via SIRT1 activation. Given that MASLD is frequently associated with abnormal glucose metabolism, further experimental validation is necessary to explore the potential effects of METTL1 gene-modified MSCs on glucose metabolism disorders in MASLD.

## Conclusions

In summary, our findings demonstrate that MSCs with METTL1 gene deficiency promote the secretion of NAMPT, which activates SIRT1 in host cells to suppress SREBP1 expression, thereby inhibiting abnormal lipid synthesis in MASLD and improving associated metabolic disorders. This research provides genetic modification targets for MSCs in the treatment of MASLD, offering valuable preclinical data for cell therapy applications.

## Supplementary Material

szag016_Supplementary_Data

## Data Availability

The datasets used and/or analyzed during the current study are available from the corresponding author on reasonable request. The RNA-seq data have been submitted to SRA with Accession number SUB15415886.
