# Supplementary material for: METTL1-deficient mesenchymal stem cells protect against metabolic-associated fatty liver disease by increasing NAMPT secretion
Source: Stem Cells Transl Med. 2026 Mar 29;15(4):szag016. doi: 10.1093/stcltm/szag016 (PMC13032905; doi:10.1093/stcltm/szag016)
Supplement: szag016_Supplementary_Data [file szag016_supplementary_data.zip › Supplementary Materials-Final.docx]

**METTL1-deficient mesenchymal stem cells protect against metabolic-associated fatty liver disease by increasing NAMPT secretion**

Jiang Du^a,b#^, Yuxuan Zhang^a,b#^, Chiheng Wang^a,b^, Yuyuan Wang^a,b^, Hongen Zhang^c^, Dunyong Zhao^d*^, Juntang Lin^a,b*^

^a^Henan Collaborative Innovation Center of Stem Cells and Biotherapy, School of Medical Engineering, Henan Medical University (Xinxiang Medical University), Xinxiang 453003, China.

^b^Henan Joint International Research Laboratory of Stem Cell Medicine, School of Medical Engineering, Henan Medical University (Xinxiang Medical University), Xinxiang 453003, China.

^c^School of pediatrics, Henan Medical University (Xinxiang Medical University), Xinxiang 453003, China.

^d^ Department of Gastroenterology, Institute of Digestive Diseases of PLA, The First Affiliated Hospital (Southwest Hospital) of Third Military Medical University (Army Medical University), Chongqing 400038, China.

Short Title:

METTL1-deficient MSCs protect against MASLD by increasing NAMPT secretion

***Corresponding author**

Dunyong Zhao

Department of Gastroenterology, Institute of Digestive Diseases of PLA, The First Affiliated Hospital (Southwest Hospital) of Third Military Medical University (Army Medical University), Chongqing 400038, China.

Email: zdy930@163.com

Juntang Lin

Henan Collaborative Innovation Center of Stem Cells and Biotherapy, School of Medical Engineering, Xinxiang Medical University, East of Jinsui Road #601, Xinxiang City, Henan Province, China

Email: [linjtlin@126.com](mailto:linjtlin@126.com)

^#^ These authors contributed equally to this work

**Table S1 The primer pairs used in this study**

*qPCR primers for human*

| Mettl1-F | GGCAACGTGCTCACTCCAA |
| --- | --- |
| Mettl1-R | CACAGCCTATGTCTGCAAACT |
| Fasn-F | CTCAGCCGCCATCTACAACA |
| Fasn-R | GCCAGCGTCTTCCACACTAT |
| Srebp1c-F | CCCTGGTCTACCATAAGCTGC |
| Srebp1c-R | CTTCACTCTCAATGCAGCCG |
| Scd1-F | AAACCTGGCTTGCTGATG |
| Scd1-R | GGGGGCTAATGTTCTTGTCA |
| Acaca-F | AGGAGCTGTC TATTCGGGGT |
| Acaca-R | GGTCGCTCAGCCTGTACTTT |
| Fads1-F | CCGACATCATCCACTCACTAAA |
| Fads1-R | AGTCTTCCTCCTCTTCTTCCA |
| NAMPT-F | GGTTCTTGGTGGAGGTTTGCTAC |
| NAMPT-R | GAAGACGTTAATCCCAAGGCC |
| SOD2-F | GTCAACCATCAAAGAGGTCTGC |
| SOD2-R | GACTGGAGATACAGGTCTTGGT |
| LDHA-F | CGAAGACAAATTGAAGGGAGAG |
| LDHA-R | CGTGATAATGACCAGCTTGGA |
| MYDGF-F | TCGTGCATTCCTTCTCCCAT |
| MYDGF-R | ACCTCTGCCTTGAACTGTGT |
| CHI3L1-F | CGTCAACACACTCAAGAACAGG |
| CHI3L1-R | TCTTGGAAAATCTTTGAGACCCA |
| PRDX4-F | GCAAAG CGAAGATTTCCAAG |
| PRDX4-R | GGCCAAATG GGTAAACTGTG |
| GLRX-F | TTGGAGCTCTGCAGTAACCAC |
| GLRX-R | CATCCACCAGAAGTGCTGTCA |
| PCSK9-F | AGGGGAGGACATCATTGGTG |
| PCSK9-R | CAGGTTGGGGGTCAGTACC |
| GAPDH-F | GGAGCGAGATCCCTCCAAAAT |
| GAPDH-R | GGCTGTTGTCATACTTCTCATGG |

*qPCR primers for mouse*

| Fasn-F | GCGGGTTCGTGAAACTGATAA |
| --- | --- |
| Fasn-R | GGGTTAGAACGTGCCAACAAGAA |
| Srebp1c-F | GGAGCCATGGATTGCACATT |
| Srebp1c-R | GGCCCGGGAAGTCACTGT |
| Scd1-F | CCGGAGACCCCTTAGATCGA |
| Scd1-R | TAGCCTGTAAAAGATTTCTGCAAACC |
| Acaca-F | GGATGACAGGCTTGCAGCTAT |
| Acaca-R | CACAGCCTATGTCTGCAAACT |
| Fads1-F | CCAGCTTTGAACCCACCAAGA |
| Fads1-R | CAGCAGGATGTGAAGCAGGTAGAC |
| GAPDH-F | TGAACGGGAAGCTCACTG |
| GAPDH-R | TCCACCACCCTGTTGCTG |


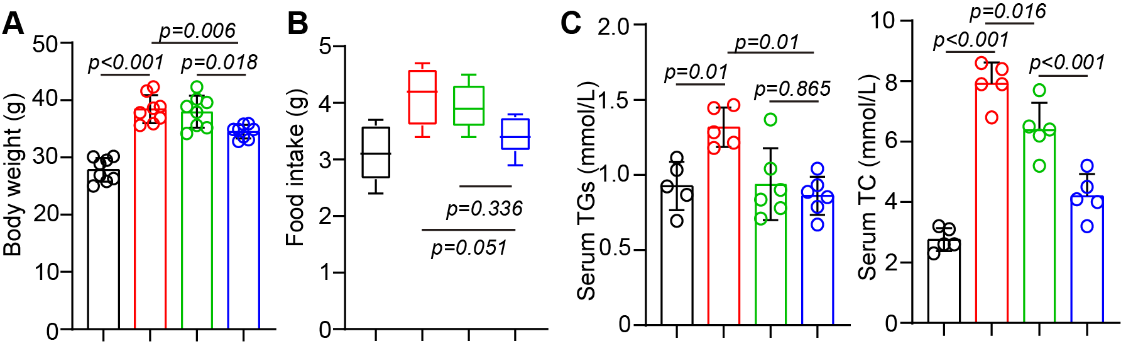


**Supplementary Fig. 1. Transplantation of METTL1-deficient MSCs alleviates metabolic disorders associated with MASLD.** (A, B) After 8 weeks of HFD feeding, mice were administered tail vein injections of PBS, MSC^shGFP^, or MSC^shMETTL1^ cells.  Following 7 weeks of cell transplantation, mouse body weight and food intake were measured. (C) Levels of TG and TC in the serum of the indicated groups were assessed. For all statistical analyses, individual data points represent individual mice, and data are presented as mean ±S.E.M. Statistical significance is indicated as shown in the figure.


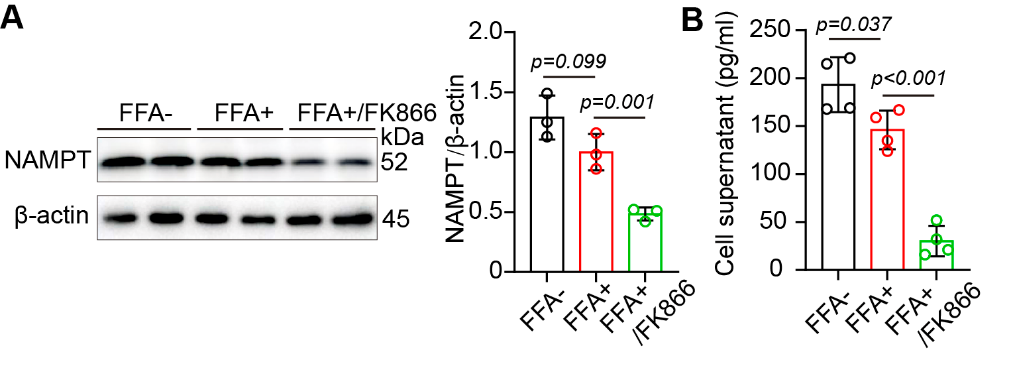


**Supplementary Fig. 2. FK866 inhibits NAMPT expression and secretion in MSCs.** (A) Western blot analysis and quantitative assessment were performed to measure NAMPT levels in MSCs treated with FK866. (B) ELISA was used to determine the secretion levels of NAMPT in FK866-treated MSCs. For all statistical graphs, data are presented as mean ± S.E.M, with statistical significance is indicated in the figure.


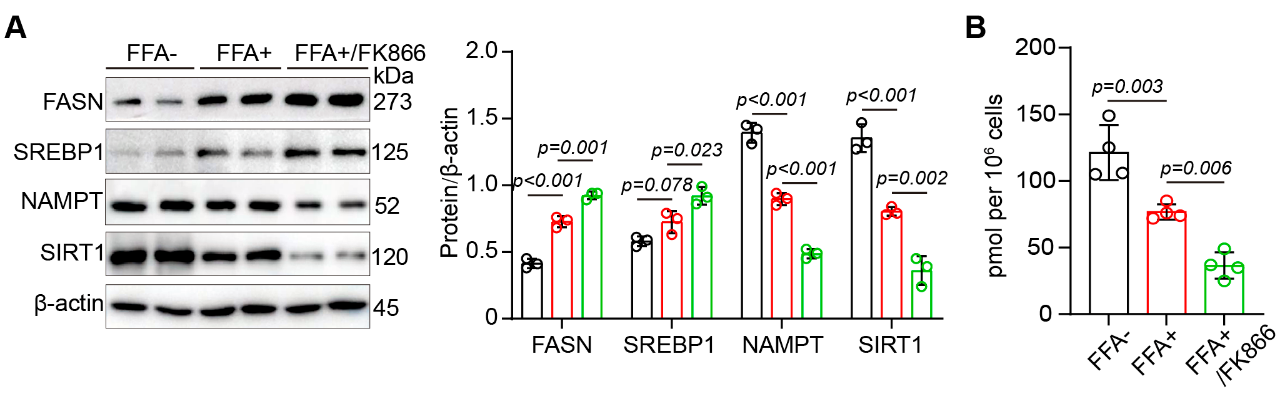


**Supplementary Fig. 3. FK866 inhibits lipid synthesis mediated by NAMPT/SIRT1/SREBP1 in hepatocytes.** (A) Western blot analysis and quantitative assessment were conducted to evaluate the expression levels of NAMPT, SIRT1, SREBP1, FASN, and SCD1 in HepG2 cells treated with FK866. (B) NAD+ content was measured in the specified cells. For all statistical graphs, data are presented as mean ± S.E.M, with statistical significance is indicated in the figure.


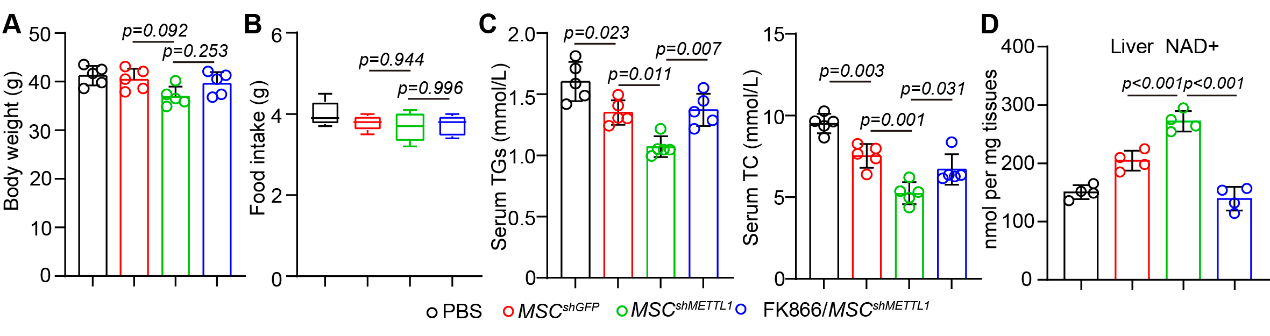


**Supplementary Fig. 4. FK866 treated MSC^shMETTL1^ transplants reduces NAD+ levels in mouse livers.** (A, B) After 8 weeks of HFD feeding, mice were administered tail vein injections of PBS, MSC^shGFP^, or MSC^shMETTL1^ cells. After 7 weeks of cell transplantation, mouse body weight and food intake were measured. (C) Serum TG and TC were conducted on the designated groups. (D) NAD+ content was measured in the livers of mice transplanted with different cell types. For all statistical graphs, data are presented as mean ± S.E.M, with statistical significance is indicated in the figure.


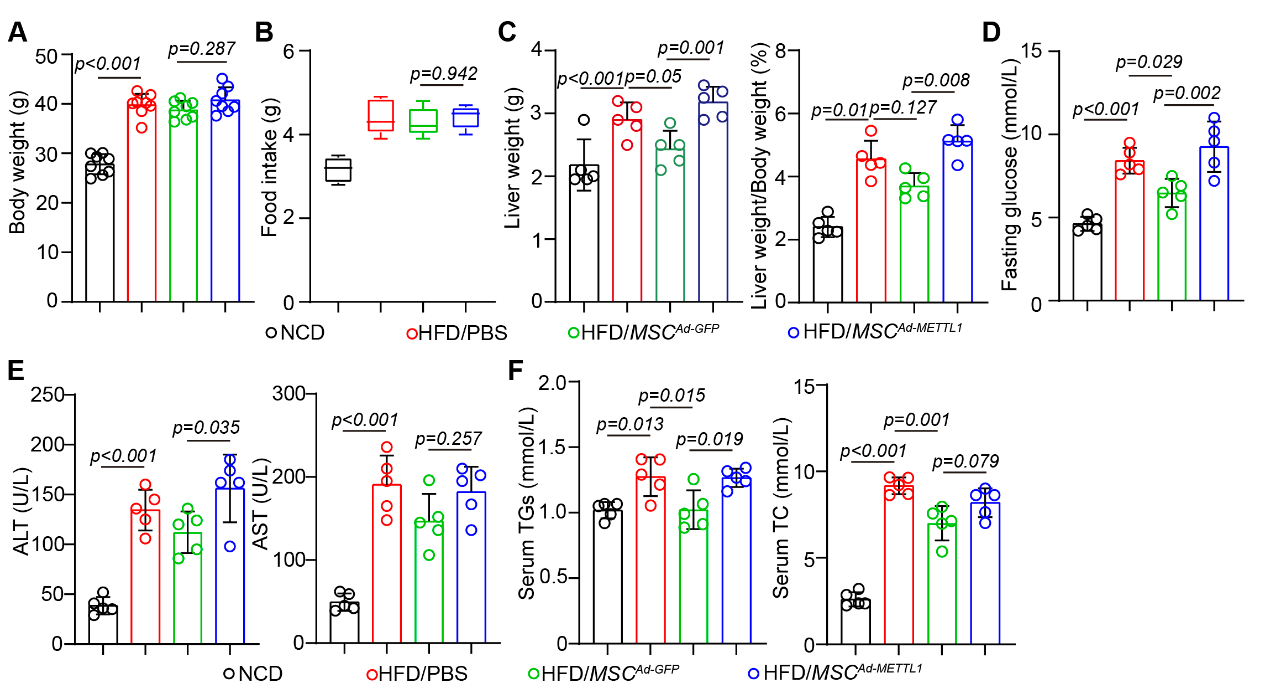


**Supplementary Fig. 5. METTL1-overexpressing MSCs accelerate metabolic disorders associated with MASLD.** (A, B) After 8 weeks of HFD feeding, mice received tail vein injections of PBS, MSC^Ad-GFP^, or MSC^Ad-METTL1^ cells. Body weight and food intake were measured 7 weeks post-cell transplantation. (C) Liver weight and the liver-to-body weight ratio were assessed in the indicated mice. (D) Fasting blood glucose levels were evaluated in the specified mice. (E) Serum ALT and AST levels were measured 7 weeks post-cell transplantation. (F) Serum TG and TC were conducted on the designated groups. For all statistical graphs, data are presented as mean ± S.E.M, with statistical significance is indicated in the figure.
