# Supplementary figures and images for: METTL1-deficient mesenchymal stem cells protect against metabolic-associated fatty liver disease by increasing NAMPT secretion
Source: Stem Cells Transl Med. 2026 Mar 29;15(4):szag016. doi: 10.1093/stcltm/szag016 (PMC13032905; doi:10.1093/stcltm/szag016)

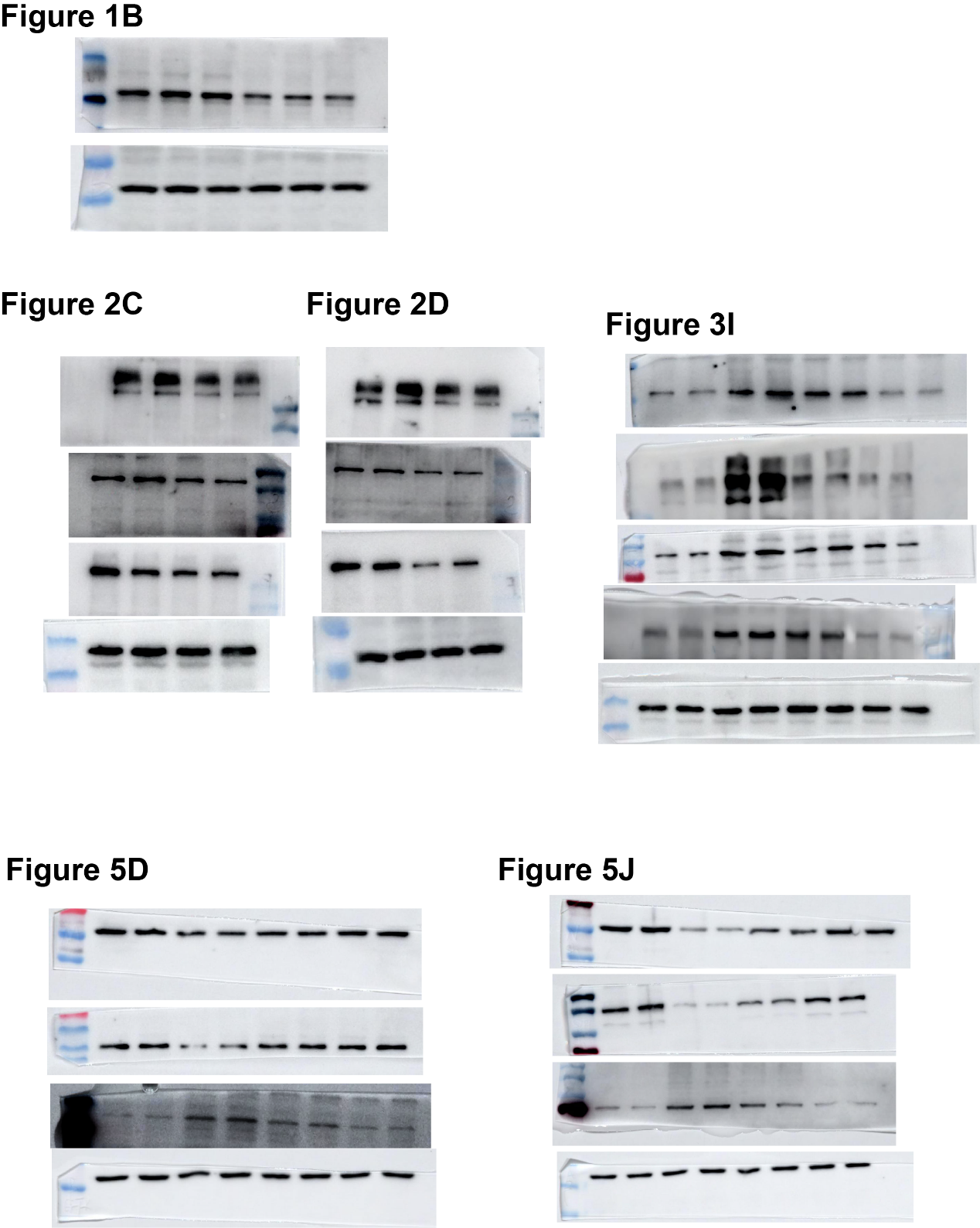

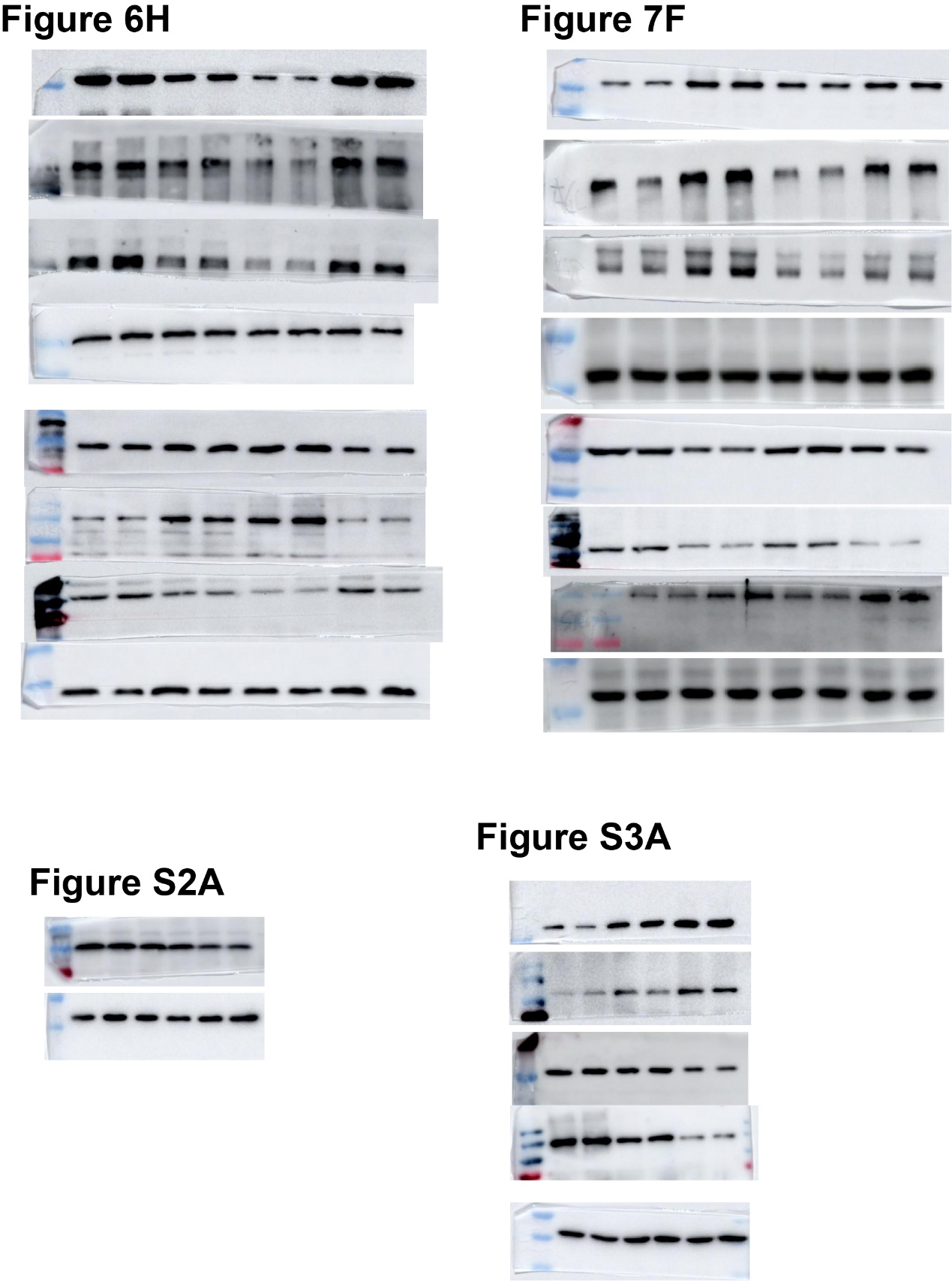

Supplement: szag016_Supplementary_Data [file szag016_supplementary_data.zip › Uncropped Western Blots-Final.docx]
